# Targeting CAMKK2 and SOC Channels as a Novel Therapeutic Approach for Sensitizing Acute Promyelocytic Leukemia Cells to All-Trans Retinoic Acid

**DOI:** 10.3390/cells10123364

**Published:** 2021-11-30

**Authors:** Faten Merhi, Karla Alvarez-Valadez, Jenifer Trepiana, Claire Lescoat, Alexis Groppi, Jean-William Dupuy, Pierre Soubeyran, Guido Kroemer, Pierre Vacher, Mojgan Djavaheri-Mergny

**Affiliations:** 1 Institut Bergonié, INSERM U1218, University of Bordeaux, 33000 Bordeaux, France; merhi.faten@gmail.com (F.M.); jenifer.trepiana@ehu.eus (J.T.); P.Soubeyran@bordeaux.unicancer.fr (P.S.); 2 PRASE, Platform of Research and Analysis in Environmental Sciences, Doctoral School of Sciences and Technologies, Lebanese University, Hadat Campus, Beirut 1003, Lebanon; 3 Centre de Recherche des Cordeliers, INSERM UMRS 1138, Sorbonne Université, Université de Paris, Équipe 11 labellisée par la Ligue Contre le Cancer, 75006 Paris, France; alvarezvaladez4@gmail.com (K.A.-V.); kroemer@orange.fr (G.K.); 4 Metabolomics and Cell Biology Platforms, Institut Gustave Roussy, 94805 Villejuif, France; 5 Centre de Bioinformatique de Bordeaux (CBiB), University of Bordeaux, 33076 Bordeaux, France; claire.lescoat@gmail.com (C.L.); alexis.groppi@u-bordeaux.fr (A.G.); 6 University of Bordeaux, CNRS, IBGC, UMR 5095, 33077 Bordeaux, France; 7 University of Bordeaux, Plateforme Protéome, 33076 Bordeaux, France; jean-william.dupuy@u-bordeaux.fr; 8 Pôle de Biologie, Hôpital Européen Georges Pompidou, AP-HP, 75015 Paris, France

**Keywords:** differentiation, cell death, autophagy, cancer, ORAI1, STIM1, AMPK, therapy, resistance to therapy

## Abstract

Calcium ions (Ca^2+^) play important and diverse roles in the regulation of autophagy, cell death and differentiation. Here, we investigated the impact of Ca^2+^ in regulating acute promyelocytic leukemia (APL) cell fate in response to the anti-cancer agent all-trans retinoic acid (ATRA). We observed that ATRA promotes calcium entry through store-operated calcium (SOC) channels into acute promyelocytic leukemia (APL) cells. This response is associated with changes in the expression profiles of ORAI1 and STIM1, two proteins involved in SOC channels activation, as well as with a significant upregulation of several key proteins associated to calcium signaling. Moreover, ATRA treatment of APL cells led to a significant activation of calcium/calmodulin-dependent protein kinase kinase 2 (CAMKK2) and its downstream effector AMP-activated protein kinase (AMPK), linking Ca^2+^ signaling to autophagy. Pharmacological inhibition of SOC channels and CAMKK2 enhanced ATRA-induced cell differentiation and death. Altogether, our results unravel an ATRA-elicited signaling pathway that involves SOC channels/CAMKK2 activation, induction of autophagy, inhibition of cellular differentiation and suppression of cell death. We suggest that SOC channels and CAMKK2 may constitute novel drug targets for potentiating the anti-cancer effect of ATRA in APL patients.

## 1. Introduction

Calcium ions (Ca^2+^) function as second messengers in a variety of cell signaling pathways and contribute to a plethora of physiological responses, including gene transcription, cell proliferation, differentiation, apoptosis and autophagy [1,2,3]. The outputs of calcium responses vary with cell type and stimuli and depend on the intensity, duration, frequency and spatiotemporal characteristics of the Ca^2+^ signals. Two major calcium entry pathways exist in non-excitable cells: (i) the store-operated calcium entry (SOCE) or capacitative Ca^2+^ entry and (ii) a Ca^2+^ store depletion-independent receptor occupation-dependent calcium entry pathway [4,5]. Calcium channels play key roles in cell homeostasis by ensuring a low cytoplasmic Ca^2+^ concentration at resting conditions, but an increase of Ca^2+^ levels in response to a variety of stressors can ignite cell proliferation, differentiation and death. The intracellular level of Ca^2+^ is also regulated through Ca^2+^-binding proteins that operate as Ca^2+^ sensors through activation of Ca^2+^ calcium signaling proteins. Calmodulin is an example of a calcium-binding protein that is involved in the initiation of a Ca^2+^ signaling cascade [6].

The endoplasmic reticulum represents a major store of intracellular Ca^2+^ in the cells that can mobilize Ca^2+^ in response to intrinsic and environmental cues [7]. Ca^2+^ accumulation in the endoplasmic reticulum (ER) is accomplished by the sarco-/ER Ca^2+^-ATPase (SERCA) pump that transports calcium from the cytosol into the ER using ATP as a source of energy. Upon depletion of the ER Ca^2+^ store, Stromal Interaction Molecule 1 (STIM1) forms oligomers at the ER membrane and then translocates to the plasma membrane to activate ORAI calcium release-activated calcium modulator 1 (ORAI1), the pore-forming subunit of SOC channels [8]. As a consequence, Ca^2+^ entry is activated, ensuring the replenishment of ER Ca^2+^ store through the stimulation of SERCA [4].

Macroautophagy (hereafter named autophagy) is a vesicular process that delivers cellular components to the lysosomes for degradation [9]. Autophagy is essential for many key physiological processes, including development, cell survival, differentiation and immune responses [10]. At the molecular level, autophagy is orchestrated by a set of autophagy-related (ATG) proteins that are regulated by several key signaling pathways, such as mammalian target of rapamycin complex 1 (MTORC1) and AMP-activated protein kinase (AMPK). A number of studies suggest that Ca^2+^ signaling controls various steps of autophagy [11]. Indeed, it was shown that calcium/calmodulin-dependent protein kinase kinase 2 (CAMKK2) and AMP-activated protein kinase (AMPK) are both involved in autophagy stimulation upon an increase of cytosolic Ca^2+^ [11,12]. Conversely, in some circumstances, it has been reported that Ca^2+^ exerts a negative control of autophagy by inducing the activation of mTOR [13]. Thus, the implication of Ca^2+^ in the regulation of autophagy is complex and context-dependent, as Ca^2+^ can either act as a positive or a negative regulator of autophagy. The complexity of intracellular calcium signaling and the cell type-dependent effect of Ca^2+^ might be responsible for the dual role of calcium in the regulation of autophagy.

All-trans retinoic acid (ATRA) is an active metabolite of vitamin A that is commonly used in the treatment of patients with acute promyelocytic leukemia (APL) [14]. However, some APL patients are refractory to ATRA or relapse after a transient response [15]. This emphasizes the need for new therapeutic strategies aiming at improving the anti-cancer effect of ATRA. We and others have previously shown that autophagy is activated during ATRA-induced granulocytic differentiation of acute promyelocytic leukemia cells [15,16]. The induction of autophagy by ATRA occurs through WIPI-dependent but BECN1-independent mechanisms [17,18]. In APL cells, ATRA also induces increased expression levels of several autophagy-regulatory transcripts [19,20,21], as well as downregulation of endogenous inhibitors of autophagy (such as BCL-2) [17,19,20,21,22]. Although the transcription regulation of autophagy by ATRA has been well-documented, little is known about how ATRA regulates autophagy at the post-translational level (e.g., phosphorylation, ubiquitination, acetylation). Given the importance of Ca^2+^ in regulating multiple steps of autophagy, we hypothesized that Ca^2+^ might be involved in the control of autophagy by ATRA in APL cells. We thus examined how ATRA-regulated Ca^2+^ fluxes might affect autophagy, differentiation and cell death in APL.

## 2. Materials and Methods

Reagents: AICA-riboside (#2627-69-2)**,** ATRA (#CR2625); adensine 5’-triphosphate (ATP) disodium salt hydrate (#34369-0708); BAPTA-AM [1,2-*bis*-(o-aminophenoxy) ethane-N,N,N′,N′-tetraacetic acid tetra-(acetoxymethyl) ester] (#126150-97-8); Benzonase endonuclease (#71205); 8-CPT-cAMP, 8-(4-chlorophenylthio) 3’,5’-cyclic adenosine monophosphate (#C3912); metformin (#PHR1084); and thapsigargin (#67526-95-8) were purchased from Sigma-Aldrich/Merck Millipore, France. STO-609 (#S8274) was purchased from Selleckchem, USA; BTP2, *N*-[4-[3,5-*Bis*(trifluoromethyl)-1*H*-pyrazol-1-yl] phenyl]-4-methyl-1,2,3-thiadiazole-5-carboxamide (#3939) and 2-APB (2-aminoethoxydiphenyl borate) (#1224) were purchased from TOCRIS Bioscience, France. Tetra-methylrhodamine methyl ester (TMRM) (#T-668) and propidium iodide (# P3566) were purchased from Life Technologies, France. Antibodies against the following proteins were used: phospho-AMPK, Thr 172 (#2531), AMPK (#2532), phospho-CAMKK2 (Ser511) (#12818), and LC3A/B (#4108) and STIM1 (#5668) were purchased from Cell Signaling Technology, USA; CAMKK2 (#ab168818) was purchased from Abcam, UK; LC3 (#L7543) and ORAI1 (# 08264) were purchased from Sigma-Aldrich, France; p62/SQSTM1 (#610832), CD11c (#333145) and CD11c (#560895) were purchased from BD biosciences, France. Cell culture media were purchased from Life Technologies-Invitrogen, France. 

### 2.1. Cell Culture

The acute promyelocytic leukemia-derived cell lines, NB4 (maturation-sensitive), and NB4-LR1 (maturation-resistant), carrying the reciprocal chromosomal translocation t(15: 17), were obtained from Dr. Michel Lanotte [23]. The human cervical cancer cells HeLa and the human leukemic T lymphoblast Jurkat cells were purchased from the American Type Culture Collection. We also used Jurkat cell lines expressing either small hairpin (sh)RNA against ORAI1 or a dominant negative mutant of ORAI1 (ORAI E106A). Both cell lines were previously generated and validated for the inhibition of SOCE-dependent Ca^2+^ entry in [24]. All cell lines used in this study were grown at 37 °C in a humidified atmosphere with 5% CO_2_ in RPMI 1640 culture media supplemented with 10% fetal bovine serum, 100 units/mL penicillin, 100 mg/mL streptomycin and 2 mM glutamine (Gibco-Life Technologies).

### 2.2. Measurement of Intracellular Ca^2+^ Concentration

Single-cell [Ca^2+^] cytosolic imaging was performed ratiometrically using Fura-calcium indicators, as previously described [24]. Briefly, NB4 cells were loaded with 1 μM Fura2-AM at room temperature for 30 min in Hank’s Balanced Salt Solution (HBSS). After washing with HBSS, the cells were incubated for 15 min in the absence of Fura2-AM to complete de-esterification of the dye. Cells were then placed in the thermostated chamber (37 °C) of an inverted epifluorescence microscope (Olympus IX70) equipped with a 64 X, UApo/340–1.15 W water-immersion objective (Olympus), and fluorescence micrograph images were captured at 510 nm and at 12-bit resolution by a fast-scan camera (CoolSNAP fx Monochrome, Photometrics). Fura2-AM was alternately excited at 340 and 380 nm, and ratios of the resulting images (excitations at 340 and 380 nm and emission filter at 520 nm) were obtained at constant intervals (5 s or 10 s according to the stimulus). Fura-2 ratio (Fratio 340/380) images were scored and the Fratio values from the regions of interest (ROIs) drawn on individual cells were monitored during the experiments and then analyzed offline with Universal Imaging software, including Metafluor and Metamorph. Each experiment was independently repeated 3 times, and for each experimental condition, we scored an average of more than 20 single-cell traces. Values are reported as mean ± SD.

### 2.3. Immunoblot Assay

After each treatment, cells were incubated in the lysis buffer Tris/SDS buffer (10 mM Tris, pH 7.4, 1% SDS) supplemented with a mix of protease (protein inhibitor cocktail tablets (#11836170001, Roche, France) and phosphatase inhibitors (PhosSTOP Easypack; #04906837001, Roche, France) and then treated with Benzonase endonuclease for 5 min at room temperature. Fractions of cell extracts containing 30 μg proteins were subjected to SDS-PAGE electrophoresis as previously described [25]. After electrophoresis, proteins were transferred to a nitrocellulose blotting membrane (#10600001, GE Healthcare, Life sciences, France). Protein loading was checked by staining the membranes with Ponceau Red S dye ((#P3504, Sigma-Aldrich, France). Membranes were then incubated with primary antibodies according to the respective manufacturer’s instruction protocols, followed by the addition of appropriate horseradish peroxidase-conjugated secondary antibody. Immuno-stained proteins were visualized on Image quant Las 4000 (GE Healthcare). The densitometry quantification was performed using the ImageJ software. All of the experiments were repeated at least two times. Representative images are shown. 

### 2.4. Label-Free Quantitative Proteomics

For this assay, three independent biological replicates were used for NB4 cells treated with ATRA (1 μM, for three days) and untreated cells. Ten µg of proteins were loaded on a 10% acrylamide SDS-PAGE gel, and proteins were visualized by Colloidal Blue staining. Migration was stopped when samples had just entered the resolving gel and the unresolved region of the gel was cut into only one segment. Proteins were reduced, alkylated and digested by trypsin, as previously described [26]. Online nanoLC-MS/MS analyses were performed using an Ultimate 3000 RSLC Nano-UPHLC system (Thermo Fisher Scientific, Waltham, MA, USA) coupled to a nanospray Q-Exactive hybrid quadrupole-Orbitrap mass spectrometer (Thermo Fisher Scientific, USA). Digested protein extracts weighing 0.5 µg were loaded on a 300 µm ID × 5 mm PepMap C_18_ precolumn (Thermo Fisher Scientific, USA) at a flow rate of 10 µL/min. The term “online” is used to indicate that there is a coupling/association/direct connection of the liquid chromatography (LC) technique with a mass spectrometer (MS): real-time analysis of the sample by LC-MS coupling. After 5 min desalting, peptides were online separated on a 75 µm ID × 25 cm C_18_ Acclaim PepMap^®^ RSLC column (Thermo Fisher Scientific, USA) with a 4–40% linear gradient of solvent B (0.1% formic acid in 80% ACN) in 108 min. The separation flow rate was set at 300 nL/min. The mass spectrometer operated in positive ion mode at a 1.8 kV needle voltage. Data were acquired using Xcalibur 3.1 software in a data-dependent mode. MS scans (*m*/*z* 350-1600) were recorded at a resolution of R = 70000 (@ *m*/*z* 200) and an AGC target of 3 × 10^6^ ions collected within 100 ms. Dynamic exclusion was set to 30 s and the top 12 ions were selected from fragmentation in HCD mode. MS/MS scans with a target value of 1 × 10^5^ ions were collected with a maximum fill time of 100 ms and a resolution of R = 17500. Additionally, only +2 and +3 charged ions were selected for fragmentation. The other settings were as follows: no sheath and no auxiliary gas flow; heated capillary temperature, 200 °C; normalized HCD collision energy of 27%; and an isolation width of 2 *m*/*z*. Protein identification and Label-Free Quantification (LFQ) were performed in Proteome Discoverer 2.4. MS Amanda 2.0, Sequest HT and Mascot 2.5 algorithms were used for protein identification in batch mode by searching against a Uniprot *Homo sapiens* database (75,091 entries, release 10 May 2020). Two missed enzyme cleavages were allowed for the trypsin. Mass tolerances in MS and MS/MS were set to 10 ppm and 0.02 Da. Oxidation (M) and acetylation (K) were searched as dynamic modifications and carbamidomethylation (C) as static modification. Peptide validation was performed using the Percolator algorithm [27] and only “high confidence” peptides were retained corresponding to a 1% false discovery rate at peptide level. Minora feature detector node (LFQ) was used along with the feature mapper and precursor ions quantifier. The normalization parameters were selected as follows: (1) unique peptides, (2) precursor abundance based on intensity, (3) normalization mode: total peptide amount, (4) protein abundance calculation: summed abundances.

### 2.5. Bioinformatic Analysis of Proteomic Data

Bioinformatic analysis was performed comparing differential protein expression levels in NB4 cells treated with ATRA (1 μM, for three days) versus untreated cells, using in-house Python scripts. Only proteins marked as “Master proteins” by Proteome Discoverer 2.4 were used in all the analyses. Out of these we discarded proteins described as “contaminant” (according to a software-specific database) and those identified with less than two unique peptides. Normalized abundance values were reduced by standard deviation of all measures per protein of a comparison. For each comparison:

(i) Proteins whose expression was measured in both conditions, expression measure reproducibility was ensured by (a) discarding proteins with more than one missing value per condition and (b) discarding proteins with a coefficient of variation $\frac{\sigma }{\mu }>0.5$ in either of two conditions (with σ the standard deviation and μ the arithmetic mean). Remaining missing values were ignored;

(ii) Proteins whose expression was measured exclusively in one condition were kept. We discarded condition-specific proteins having one or more missing values in the condition in which they were identified to ensure the reliability of the identification.

For each protein, fold change was computed as the ratio of geometrical means between triplicates of expression values. Differential analysis between conditions was performed by first identifying the probability distribution corresponding to the best fit for the z-scores of the ratios and then identifying the outliers. The best distribution was defined as the distribution minimizing sum of square error (SSE). *p*-values were computed from the cumulative distribution function of the chosen distribution with a two-tailed test. For condition-specific proteins, the *p*-value was set to 0 (and fold change to 1000) to reflect that they were specifically retrieved in one condition. The *p*-value significance threshold was set to 0.05.

### 2.6. Cell Death Measurement

Cell death was determined by measuring plasma membrane permeability and mitochondrial transmembrane permeability using propidium iodide dye and TMRM dye, respectively. Briefly, supernatant and attached cells were collected, pelleted at 350 g for 5 min and loaded with either 1 μg/mL propidium iodide for 15 min or 150 nM TMRM for 30 min at 37 °C. Cells were then analyzed by flow cytometry using either a BD FACS Calibur (Becton Dickinson, Franklin Lakes, NJ, USA) or a Miltenyi Biotec instrument (Bergisch Gladbach, Germany). 

### 2.7. Evaluation of Granulocytic Differentiation

ATRA-induced granulocyte differentiation of NB4 cells was determined as previously described [19] by (i) morphological assessment of cells stained with May-Grunwald Giemsa (MGG) and (ii) analysis of the expression of the CD11c cell surface marker by flow cytometry upon discarding dead cells, followed by staining cells with propidium iodide. Results were expressed as the median of the fluorescence intensity (MFI) and were given as means plus or minus SD of at least three independent experiments.

### 2.8. Statistical Analysis

Unless otherwise stated, one-way ANOVA complemented by the Tukey test was used for the calculation of *p*-values for comparisons between different groups using Prism software. Results were given as means plus or minus SD of at least three independent experiments. *p*-values of 0.05 were considered to be statistically significant. (*p* value * < 0.05, *p* value ** < 0.01, *p* value *** < 0.001, ns: nonsignificant).

## 3. Results

### 3.1. ATRA Activates the Capacitative Ca^2+^ Entry into Acute Promyelocytic Leukemia Cells

**Figure 1:**
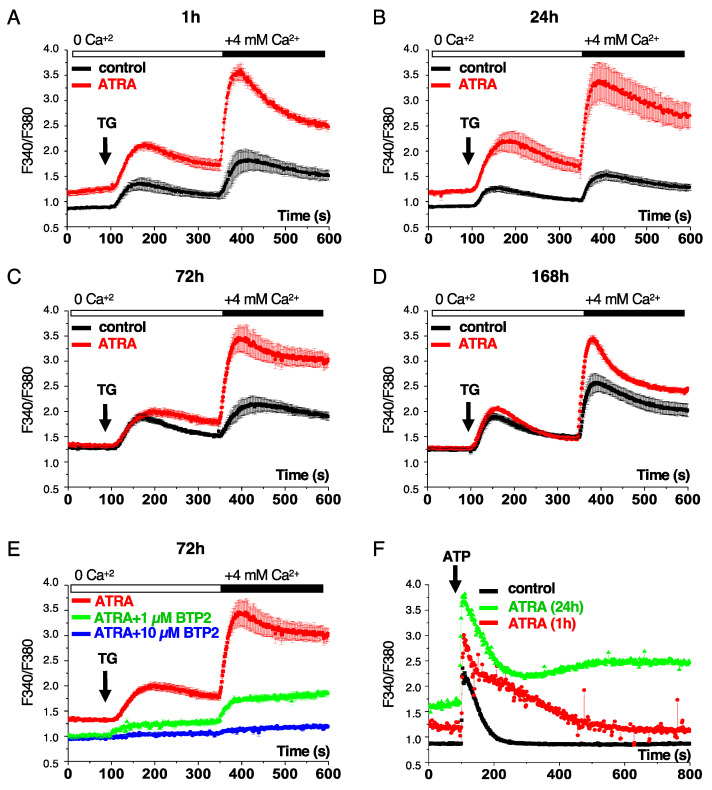
ATRA triggers Ca^2+^ responses in NB4 cells. (**A**–**D**). NB4 cells were treated with 1 μM ATRA for the indicated time. At the end of the treatment, cells were loaded with 1μM Fura 2-AM and subjected to Ca^2+^ imaging, as described in the Materials and Methods section. The relative intracellular ([Ca^2+^]i) was determined by scoring the fluorescence ratio of excitation wavelengths at 340 and 380 (R = F340/F380). Thapsigargin (100 nM) was added to evaluate ER Ca^2+^ content. The capacitative Ca^2+^ entry was scored after the addition of 4 mM Ca^2+^. (**E**) NB4 cells were pre-treated for 2 h with either 1 μM or 10 μM *N*-(4-(3,5-*bis*(tri uoromethyl)-1H-pyrazol-1-yl)phenyl)-4-methyl-1,2,3-thiadiazole-5-carboxamide (BTP2) and then treated with 1 μM ATRA for 72 h. Intracellular ([Ca^2+^]i) were recorded as described in (**A**–**D**,**F**) ATP (100 μM) was added to evaluate ER Ca^2+^ content in NB4 cells treated with 1 μM ATRA for either 1 h or 24 h. Cells were then subjected to Ca^2+^ imaging, as described in the Materials and Methods section. Data represent means ± the SD of three independent experiments (>30 cells).

To evaluate the cytosolic Ca^2+^ levels in APL cells treated with ATRA, we used thapsigargin (TG), an inhibitor of SERCA that provokes depletion of ER Ca^2+^ storage (indicative of ER Ca^2+^ content) in a Ca^2+^-free saline medium and then added 4 mM Ca^2+^ to stimulate SOCE-mediated capacitative Ca^2+^ entry. As shown in Figure 1A–D, ATRA treatment of APL patient-derived NB4 cells results in an increase in the ER Ca^2+^ content and the consequent capacitative Ca^2+^ entry in a time-dependent manner. Both responses, however, dropped after treatment of NB4 cells with ATRA for 7 days, suggesting a feedback loop regulation of the Ca^2+^ responses (Figure 1D). 

The activation of SOCE by ATRA was further verified by the use of *N*-(4-(3,5-*bis*(tri uoromethyl)-1H-pyrazol-1-yl)phenyl)-4-methyl-1,2,3-thiadiazole-5-carboxamide (BTP2), an inhibitor of SOC channels [28]. As shown in Figure 1E, the addition of BTP2 to ATRA completely inhibited ATRA-mediated Ca^2+^ entry, suggesting that ATRA-mediated Ca^2+^ entry through an SOCE-dependent mechanism. We also evaluated Ca^2+^ entry in Jurkat T cells after ATRA treatment. As shown in Supplementary Data 1A,B, treatment of Jurkat cells with ATRA led to Ca^2+^ entry through an SOCE-dependent mechanism and this response was significantly attenuated in Jurkat cells expressing a dominant negative mutant of ORAI1 (ORAIE106A), as well as in cells in which ORAI1 was knocked down by an shRNA [24]. Together, these data support the notion that ATRA-mediated Ca^2+^ entry is not restricted to NB4 cells but may represent a general cellular response. Next, we used ATP as an alternative to TG to deplete ER Ca^2+^ stores and to examine Ca^2+^ entry in NB4 cells treated with ATRA. Indeed, the binding of ATP to purinergic receptors causes the production of IP_3_ (inositol triphosphate), resulting in the depletion of ER Ca^2+^ due to the binding of IP_3_ to the IP_3_ receptors on the ER plasma membrane [7]. As expected, ATP treatment of NB4 cells resulted in a rapid and significant increase of cytosolic Ca^2+^ levels that were further restored after 200 s (Supplementary Data 1C). The stimulation of Ca^2+^ entry by ATP was completely inhibited by the IP_3_R antagonist 2-aminoethoxydiphenyl borate (2-APB) that, when used at high concentrations, functions as an SOCE inhibitor [28]. When this assay was performed in NB4 cells treated with ATRA, Ca^2+^ entry induced by ATP was significantly increased as early as one hour after treatment and remained elevated at 24 hours compared to untreated cells, again confirming the ability of ATRA to promote a rapid and persistent stimulation of capacitative Ca^2+^ entry into NB4 cells (Figure 1F). Of note, the addition of 2-ABP to ATRA markedly attenuated Ca^2+^ entry, corroborating the involvement of SOC channels in this response (Supplementary Data 1). 

From the Ca^2+^ imaging experiments (Figure 1A–D), we also noticed an increase of the basal intracellular Ca^2+^ concentration ([Ca^2+^]i) in NB4 cells treated with ATRA. This effect was observed as early as one hour after treatment (Figure 1A) and remained increased after 24 h as compared to untreated cells (Figure 1B). For longer cell culture time points (72 h to 168 h), the level of basal ([Ca^2+^]i) was increased to a comparable extent in untreated and ATRA-treated cells (Figure 1C,D).

To determine if ATRA drives Ca^2+^ mobilization from extracellular origins or intracellular sources, cells were treated with ATRA and intracellular Ca^2+^ levels were immediately measured in the presence or absence of extracellular Ca^2+^ (Supplementary Figure S1E,F). As shown in Supplementary Figure S1E, in the presence of extracellular Ca^2+^, ATRA treatment of NB4 cells led to a continuous increase in [Ca^2+^]i. However, in the absence of extracellular Ca^2+^, ATRA failed to increase [Ca^2+^]i in NB4 cells (Supplementary Figure S1F), indicating that ATRA mainly targets the entrance of Ca^2+^ from the extracellular medium rather than the release of intracellular Ca^2+^ pools. 

Interestingly, ATRA-induced Ca^2+^ responses in NB4 cells were associated with an increase in microtubule-associated protein 1 light chain 3-II (LC3-II) levels (a marker of autophagosomes) and stimulation of granulocyte differentiation (Supplementary Data Figure S2A,B), as revealed by increased nuclear lobulation and decrease nuclear cytoplasmic surface (as detected by May–Grünwald–Giemsa staining). 

### 3.2. Regulation of Ca^2+^-Regulatory Proteins by ATRA

**Figure 2:**
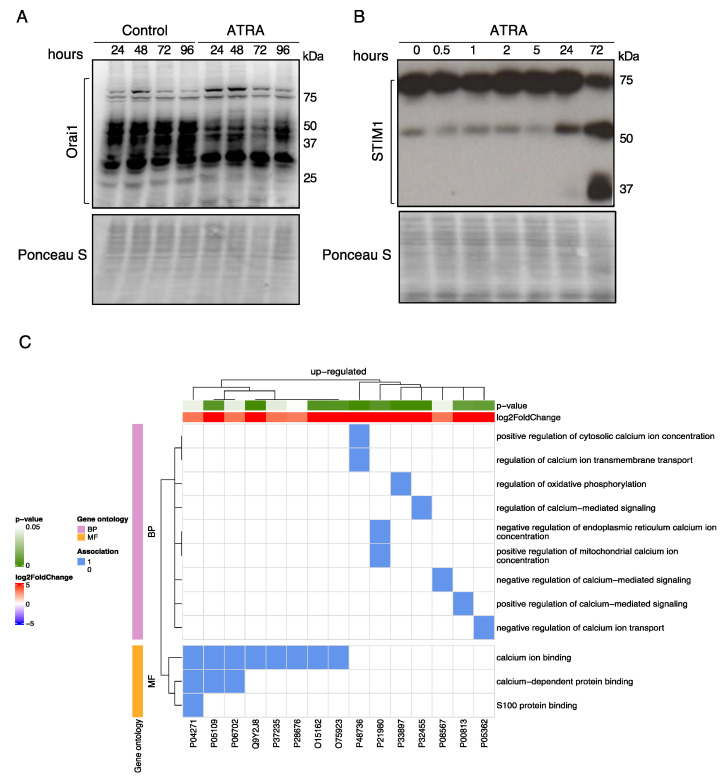
ATRA modulates calcium-regulatory proteins. (**A,B**) NB4 cells were treated with 1 μM ATRA for the indicated time. Immunoblot analyses of ORAI1 and STIM1 protein expression levels are shown. For each membrane, Ponceau S Red staining was used as protein loading control. Bands corresponding to different forms of ORAI1 and STIM1 are depicted. (**C**) NB4 cells were treated with 1 μM ATRA for 3 days and then subjected to proteomic analysis. The heat map visualizes the annotation of statistically significant proteins (*p*-value < 0.05) identified by proteomics data analysis (columns) by GO terms associated with Ca^2+^ signaling (lines; see also Supplementary Data File 3). The blue color represents an existing annotation while the white color represents none.

Next, we examined if ATRA-mediated Ca^2+^ entry is related to changes in the expression levels of Ca^2+^-regulatory proteins. First, we examined the protein expression levels of ORAI1 and STIM1 during ATRA treatment of NB4 cells, as post-translational regulations (i.e., deglycosylation, cleavage) of these proteins have been shown to influence SOCE activation [29,30]. Indeed, deglycosylation of ORAI1 reportedly stimulates SOCE, whereas the cleavage of STIM1 by calpain reduces STIM1 activity [29,30]. Immunoblotting of ORAI1 in NB4 control cells (Figure 2A) revealed several bands corresponding to distinct molecular masses (ranging from 75 kDa to 25 kDa) (Figure 2A). These bands are attributed to different glycosylated states of ORAI1 (bands of ~50 kDa are glycosylated forms of ORAI1, while ~33 kDa and ~25 kDa represent deglycosylated forms) [29]. ATRA promoted a significant deglycosylation of ORAI1 as early as one day after treatment. Immunoblot detection of STIM1 unraveled a noticeable ATRA-induced decrease in STIM1 protein (~75 kDa) levels associated with the appearance of STIM1 fragments (~50 kDa and ~37 kDa) (Figure 2B) after 72 h of treatment (Figure 2B). Altogether, these results suggest that ATRA affects the activity of SOC channels.

To gain more insight into the impact of ATRA on Ca^2+^-relevant proteins, we performed mass spectrometry to identify proteins differentially expressed in ATRA-treated NB4 cells versus untreated cells. For this analysis, we particularly focused on the expression levels of proteins associated with Ca^2+^ signaling pathways according to established gene ontology (GO) terms [31]. Only proteins the expression of which changed by a factor ≥2 (with a *p*-value < 0.05) were considered. This bioinformatic analysis led to the identification of 15 different proteins belonging to 172 GO terms (Supplementary Data 3) that were markedly upregulated in response to ATRA (Table 1).

The identified proteins belong to twelve different GO terms related to Ca^2+^ signaling. Seven of them are associated with GO biological process (BP) terms linked to positive regulation of cytosolic calcium ion concentration (GO: 0007204), regulation of calcium ion transmembrane transport (GO:1903169), regulation of oxidative phosphorylation (GO:0002082), regulation of calcium-mediated signaling (GO:0050848), negative regulation of endoplasmic reticulum calcium ion concentration (GO:0032471), positive regulation of calcium ion transmembrane transport (GO: 1903169) and negative regulation of calcium ion transport (GO:0051926). Eight proteins are associated with GO molecular function (MF) terms linked to calcium binding (GO: 0005509), calcium-dependent protein binding (GO: 0048306) and S100 protein binding (GO:441548). Altogether, these findings suggest that ATRA-mediated Ca^2+^ responses are implicated in diverse biological processes and functions in NB4 cells (Figure 2C).

### 3.3. ATRA Induces Autophagy through a Ca^2+^-Dependent Mechanism

**Figure 3:**
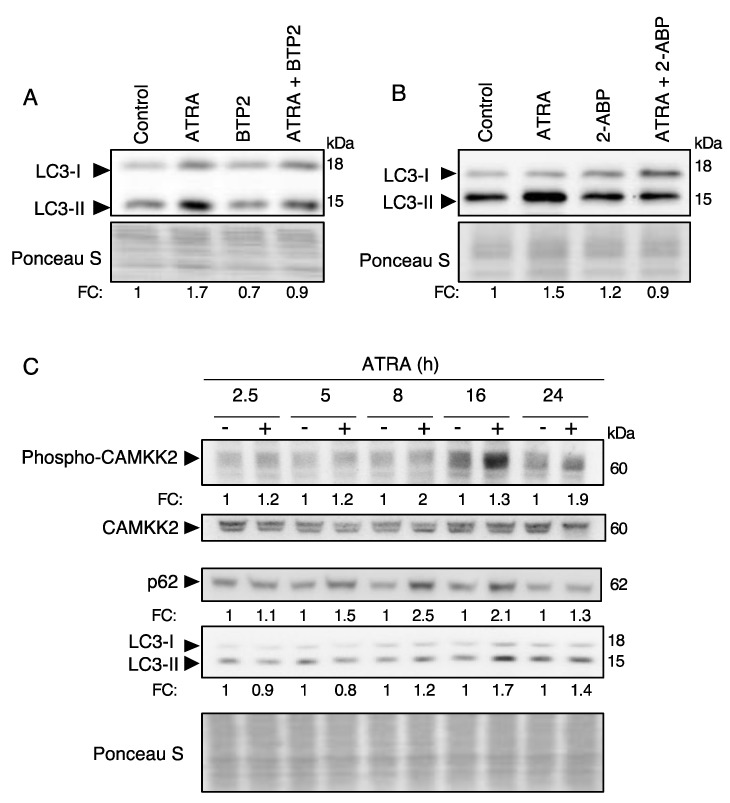
ATRA induces autophagy through a Ca^2+^-dependent mechanism. (**A**) NB4 cells were pre-treated with 5 μM BTP2 for 2 h before treatment with 1 μM ATRA for 24 h. Cell extracts were then analyzed for LC3 levels by western blotting. (**B**) Immunoblot analysis of LC3 levels in NB4 cells pre-treated with 44 μM 2-APB for 2 h and then treated with 1 μM ATRA for 24 h. (**C**) NB4 cells were treated with 1 μM ATRA for the indicated time then subjected to immunoblot analysis for phospho-CAMKK2, CAMKK2, p62 and LC3-II. Ponceau S red staining was used to score the protein loading levels. The expression levels of p62 and LC3-II were scored and normalized to the protein loading levels of each sample. The expression levels of phospho-CAMKK2 and CAMKK2 were quantified and given as ratio of phospho-CAMKK2/CAMKK2. The quantification values represent the ratio of treated cells versus untreated cells (fold change, FC) for each time point.

We and others have previously shown that ATRA promotes autophagy in APL cells [15,16,17,18,19,21,22], but so far information on the upstream regulators of autophagy remains scare. Since the stimulation of Ca^2+^ entry is an early response induced by ATRA, we reasoned that Ca^2+^ signaling might be involved in autophagy regulation. To test this hypothesis, we first examined the effect of two inhibitors of the Ca^2+^ entry (induced by SOC channels), BTP2 and 2-APB, on the accumulation of the autophagy marker LC3-II. As shown in Figure 3A,B, both inhibitors markedly attenuated the accumulation of LC3-II in NB4 cells exposed to ATRA, suggesting that SOC channel activation contributes to ATRA-induced autophagy. Similarly, chelation of intracellular Ca^2+^ by 1,2-Bis(2-aminophenoxy) ethane-N,N,N′,N′-tetraacetic acid tetrakis(acetoxymethyl ester) (BAPTA-AM) markedly reduced ATRA-induced accumulation of LC3-II confirming the contribution of Ca^2+^ signaling to autophagy regulation (Supplementary Data 2).

To elucidate the Ca^2+^ cascade involved in autophagy regulation, we next examined the activity of CAMKK2, which is the primary Ca^2+^ sensor that relays the Ca^2+^ signal to autophagy [32,33,34]. As shown in Figure 3C, ATRA treatment of NB4 cells resulted in a significant and time-dependent activation of CAMKK2, as indicated by the detection of the active phosphorylated form of CAMMK2. This response is associated with an increase in the levels of LC3-II and p62/Sequestosome 1 (SQSMT1). 

### 3.4. ATRA Promotes AMPK Activation through a Ca^2+^ -Dependent Mechanism in Differentiation-Sensitive NB4 Cells but Not in Differentiation-Resistant NB4-LR1 Cells

**Figure 4:**
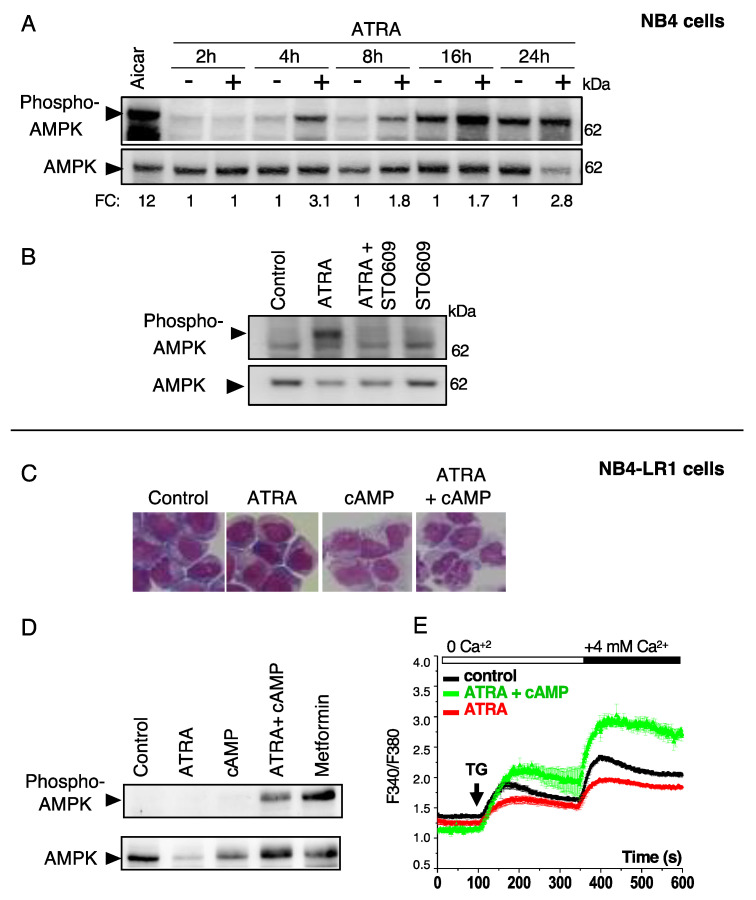
ATRA triggers AMPK activation in NB4 cells but not in NB4-LR1 cells. (**A**) Immunoblot analysis of phospho-AMPK and AMPK in NB4 cells treated with either an AMPK activator (AICAR, 500 μM, 2 h) or 1 μM ATRA for the indicated time. The expression levels of phospho-AMPK and AMPK were quantified and given as ratios of phospho-AMPK/AMPK relative to untreated cells. (**B**) Immunoblot analysis of the expression levels of phospho-AMPK and AMPK in NB4 cells pre-treated with or without STO609 (5 μg/mL) and then treated with 1 μM ATRA for 16h. (**C–E**) Maturation-resistant NB4-LR1 cells were treated with 1 μM ATRA and 100 μM 8-CPT-cAMP alone or in combination for indicated times. (**C**) Granulocytic differentiation was determined by morphological changes of cells stained with May–Grünwald–Giemsa after 3 days of treatments. (**D**) Immunoblot analysis of phospho-AMPK and AMPK proteins are shown. Metformin (2 mM, 2 h) was used as an AMPK activator. (**E**) Ca^2+^ release and entry amplitudes were scored after 7 days of treatment as described in Figure 1.

One of the best-characterized downstream targets of CAMKK2 is AMP kinase (AMPK), which plays a central role in several key processes, such as autophagy and metabolism [35]. Therefore, we investigated whether AMPK is activated by ATRA and whether this regulation relies on Ca^2+^ signaling. As shown in Figure 4A, ATRA treatment of NB4 caused a significant and time-dependent activation of AMPK, as revealed by an increase in the levels the phosphorylated form (at residue Thr 172) of this protein. Moreover, we found that STO-609, an inhibitor of CAMKK2, significantly reduced ATRA-induced AMPK activation, suggesting that CAMKK2 is involved in ATRA-driven AMPK activation (Figure 4B). Similar responses were observed in HeLa cells subjected to ATRA treatment, indicating that the activation of the CAMKK2/AMPK axis may represent a general mechanism induced by ATRA (Supplementary Figure S4).

To determine the interrelation between Ca^2+^/AMPK activation and granulocytic differentiation induced by ATRA, we next evaluated capacitative Ca^2+^ entry and AMPK activation in ATRA-treated NB4-LR1 cells, which are resistant to differentiation. Of note, the resistance to ATRA can be reverted in NB4-LR1 cells by a combination of ATRA and 8-(4-chlorophenylthio), 5’-cyclic adenosine monophosphate (8-CPT-cAMP), as evidenced by morphological changes (Figure 4C). Unlike NB4 cells, no activation of AMPK (Figure 4D) and no induction of capacitative Ca^2+^ entry (Figure 4E) was observed in differentiation-resistant NB4-LR1 cells upon ATRA treatment. Interestingly, when differentiation was reestablished in NB4-LR1 cells by the simultaneous addition of 8-CPT-cAMP and ATRA, NB4-LR1 cells exhibited significant increases in both AMPK activation and Ca^2+^ entry which were comparable to ATRA-treated differentiation-sensitive NB4 cells (Figure 4D,E). This suggests that the status of APL cell differentiation impacts the Ca^2+^ responses and AMPK activity in these cells. In addition, we previously reported that ATRA promotes autophagy in NB4 cells but not in NB4-LR1 [19], suggesting that autophagy and differentiation are interconnected processes. Altogether, these data revealed that the Ca^2+^/AMPK axis and autophagy are both activated during granulocytic differentiation of APL cells.

### 3.5. Role of Ca^2+^ Responses on ATRA-Induced Granulocytic Differentiation of APL Cells

**Figure 5:**
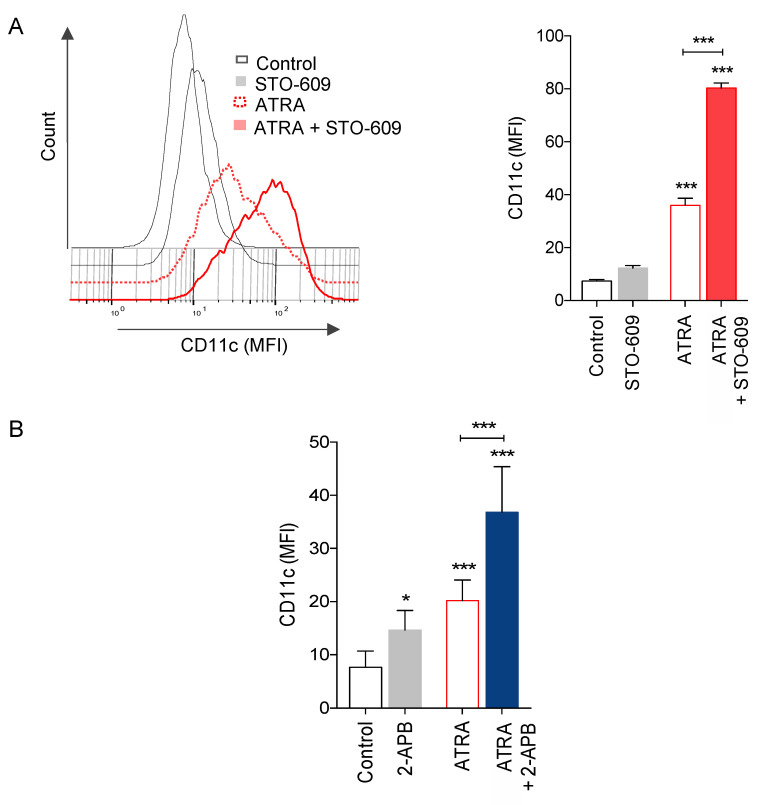
Pharmacological inhibition of SOC channels and CAMKK2 enhanced ATRA-induced cell death. (**A**) NB4 cells were pre-treated with 5 μg/mL STO609 and then treated with 1 μM ATRA for 72 h. Granulocytic differentiation was determined by cytofluorometric evaluation of CD11c expression. Left panel, representative flow cytometry histograms are shown; right panel, the median fluorescence intensity (MFI) of CD11c was scored for each sample. (**B**) NB4 cells were pre-treated with 44 μM 2APB and then treated with 1 μM ATRA for 72 h and granulocytic differentiation was determined. Results correspond to the means ± SD of three independent experiments realized in triplicate. *p* value * < 0.05, *p* value *** < 0.001.

The association between CAMKK2/AMPK pathway activation and APL cell maturation by ATRA prompted us to investigate the effects of CAMKK2/AMPK pathway inhibition on granulocyte differentiation. For this, we first evaluated differentiation of NB4 cells after 72 h of ATRA treatment in the presence or absence of the CAMKK2 inhibitor STO-609. As shown in Figure 5A, the addition of STO-609 significantly enhanced granulocytic differentiation induced by ATRA as indicated by an increase of cells expressing high levels of CD11c, a cell surface granulocyte marker.

We also examined the possible role of SOC channels on NB4 cell maturation induced by ATRA. Upon inhibition of SOC channels by 2-APB, we observed a significant increase in ATRA-induced NB4 cell maturation as indicated by the induction of CD11c expression after 72 h of treatment (Figure 5B). 

Altogether, these results indicate that the activation of both CAMKK2 and SOC channels led to the attenuation of ATRA-induced NB4 differentiation. 

### 3.6. Impact of Calcium Responses on ATRA-Induced APL Cell Death

**Figure 6:**
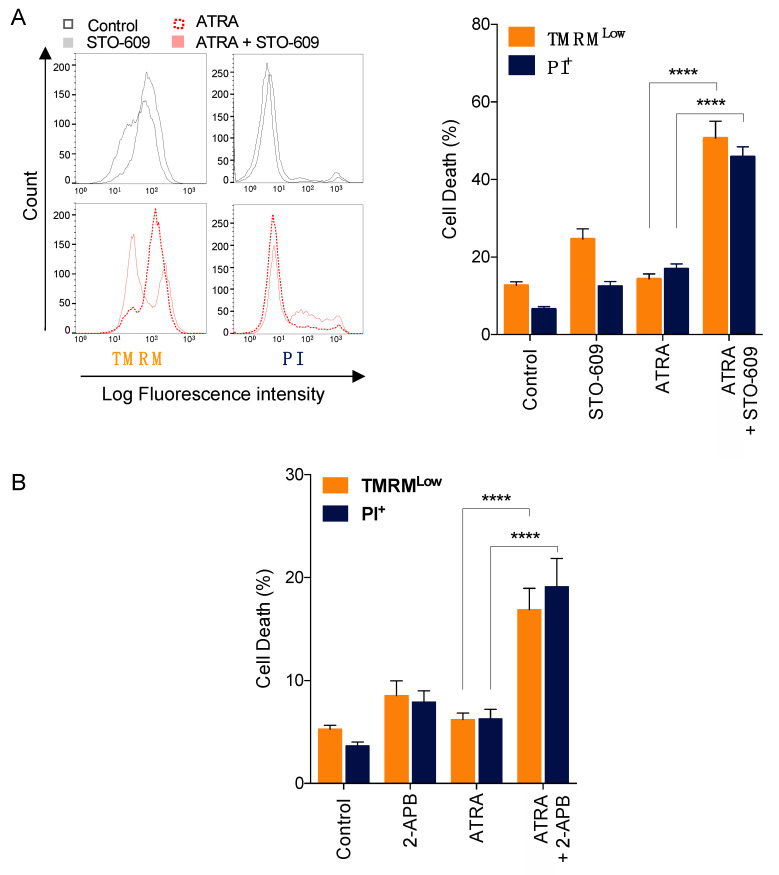
Pharmacological inhibition of SOC channels and CAMKK2 promotes ATRA-induced cell death. NB4 cells were pre-treated with STO609 (5 μg/mL) and then treated with 1 μM ATRA for 5 days. Cell death was assessed by the measurement of (i) the mitochondrial transmembrane potential using TMRM dye and (ii) the plasma membrane permeability using propidium iodide (PI) staining. (**A**) Left panel, representative flow cytometry histograms for TMRM and PI cell staining. Right panel, percentage of cells exhibiting low TMRM staining and high PI staining were scored for each condition. (**B**) NB4 cells were pre-treated with 2-APB (44 μM) prior to treatment with 1 μM ATRA for 2 days. At the end of this treatment, cell death was scored as described in (**A**,**B**) Results are means ± SD of three and four independent experiments realized in triplicate for STO-609 and 2-APB, respectively. **** *p*-value < 0.0001.

We next investigated the potential effect of CAMKK2 and SOC channel activation on cell death induced by ATRA in NB4 cells, by means of staining with the mitochondrial transmembrane potential-sensitive dye tetramethylrhodamine (TMRM) and the vital dye propidium iodide (PI). As shown in Figure 6A, inhibition of CAMKK2 activation by STO-609 significantly enhanced the induction of early (TMRM^low^ population) and late apoptosis (PI^high^ population) induced by ATRA, as evidenced by the loss of mitochondrial transmembrane potential and the permeabilization of the plasma membrane, respectively. Similarly, inhibition of SOC channels by 2-APB promoted a significant increase in early and late apoptosis induced by ATRA (Figure 6B). Altogether, these data indicate that inhibition of either CAMKK2 or SOC channels enhanced ATRA-induced cell death, suggesting that this pathway plays a pro-survival role in ATRA treated NB4 cells. 

## 4. Discussion

The role of Ca^2+^ in the control of cell death and differentiation has been documented in several settings [1,2,7], but little is known about how Ca^2+^ regulates APL cell fate in the context of ATRA treatment. Here, we report the activation of both SOC-dependent calcium entry and CAMKK2 activation by ATRA in APL-derived NB4 cells. Moreover, we demonstrated that CAMKK2 and SOC channels exert a negative feedback control on the differentiation and apoptosis of NB4 cells treated by ATRA. These results suggest that the association of CAMKK2 and SOC inhibitors with ATRA may represent a novel approach for improving the treatment of APL patients.

Our data are in line with earlier works showing that HL60 leukemia cells exhibit an increase in intracellular Ca^2+^ entry in response to several differentiating inducers (i.e., the chemotactic peptide-FMLP and the mitogen Con-A [36], retinoic acid, 1 alpha, 25 dihydroxy vitamin D3 and dimethyl sulfoxide [37] and A23187 plus retinoic acid [38]). Moreover, Launey et al. showed that ATRA induces an upregulation of SERCA3 [39] and that the SERCA activity fine-tunes the sensitivity of cells to the differentiation-inducing activity of ATRA [40].

Here, we have provided the first evidence that Ca^2+^ entry induced by ATRA occurred through a SOC-dependent mechanism. Thus, pharmacological and genetic inhibition of SOC channels markedly attenuated the ATRA-elicited Ca^2+^ entry. Post-translational regulations of ORAI1 and STIM1 expression protein levels have been shown to influence SOCE activation and calcium signaling [29,30]. We found that ATRA-induced Ca^2+^ entry was accompanied by changes in the expression patterns of ORAI1 (from glycosylated forms toward deglycosylated forms) [29] and induction of STIM1 cleavage [30]. Dörr and co-workers previously showed that the glycosylation of ORAI1 was cell-specific and that the deglycosylation of ORAI1 increased SOCE, suggesting an inhibitor effect of glycosylation of ORAI1 in calcium flux. Whether such modifications influence SOCE activity and calcium signaling induced by ATRA deserves to be investigated in more detail in future studies. 

Moreover, we found that ATRA treatment of NB4 cells resulted in the upregulation of several key proteins involved in calcium signaling, highlighting an important modulation of calcium-regulatory proteins and their associated signaling pathways by ATRA. Further studies are needed to elucidate the precise network of calcium regulation in APL cells treated with ATRA.

More importantly, we found that ATRA-induced SOCE activation contributes to the induction of autophagy and identified the CAMKK2/AMPK pathway as a sensor of this regulation. These effects were observed in differentiation-sensitive APL cells (NB4 cells) cells but not in differentiation-resistant APL cells (NB4-LR1 cells). Interestingly, when the resistance to ATRA was reverted in NB4-LR1 cells by the combined action of ATRA and 8-CPT-cAMP, calcium entry and AMPK stimulation were both restored in these cells. Moreover, the induction of autophagy by ATRA was established upon treatment with ATRA plus 8-CPT-cAMP, as shown in our previous study [19]. Altogether, these findings underscore the tight molecular links among ATRA-induced calcium entry, autophagy and differentiation. In accordance with these findings, SOC channels have been shown to positively regulate autophagy in endothelial progenitor cells through the CAMKK2- pathway [41]. On the contrary, other studies considered that SOC channel activation acts as an autophagy inhibitor, pointing to a dual role for SOC channels in regulating autophagy, perhaps depending on cell type and specific downstream Ca^2+^ signaling activated in response to a given stimuli [11,42,43,44]. Undoubtedly, further investigations will be needed to determine the exact mechanism through which SOC channels and CAMKK2 regulate ATRA-induced autophagy.

Beyond autophagy, CAMKK2 is known to regulate several other biological processes, including development, tissue remodeling, differentiation and cell death [6]. In particular, CAMKK2 activation has been shown to contribute to cell death resistance and cell survival in the context of cancer [45,46,47]. In line with these results, our data revealed that CAMKK2 activation by ATRA operates as an anti-apoptotic response in APL cells. Whether autophagy contributes to this anti-apoptotic effect remains an open question.

Depending on the cellular context and stimuli, CAMKK2 activation can exert either an inhibitory or stimulatory effect on cellular differentiation. In this regard, it has been shown that CAMKK2 activation inhibits myoblast [48] and preadipocyte differentiation [49], whereas it accelerates monocyte differentiation induced by colony stimulating factor 1 [50]. Our findings reveal that CAMKK2 suppresses granulocytic differentiation induced by ATRA. This result corroborates previous data showing the inhibitory effect of CAMKK2 on granulocytic fate commitment and differentiation in early myeloid progenitors [51].

Although in a different context, our results are consistent with previous observations showing an anti-apoptotic role for SOCE in human cancer cell lines. For example, it has been shown that human myeloid leukemia cells and prostate cancer cell lines are highly sensitive to the cytotoxic effect of SOCE antagonists [43,52]. The anti-apoptotic effect of SOCE was also shown in response to anti-cancer drugs, as exemplified by rituximab acting on B lymphoma cells and 5-fluorouracil (5-FU) or gemcitabine acting on pancreatic adenocarcinoma cells [53,54]. Conversely, SOCE activation was shown to contribute to cell death processes, particularly under oxidative conditions [55,56]. Thus, SOCE plays a subtle role in regulating apoptosis, depending on other stimuli, the cell type, as well as the intensity, duration, frequency and nature of Ca^2+^ signals.

## 5. Conclusions

In sum, we identified the SOC channels/CAMKK2 axis as a novel pathway induced by ATRA that links Ca^2+^ signaling to autophagy, cell death and differentiation. We propose that inhibiting CAMMK2 and SOC-channels may constitute a novel approach to increasing the efficacy of ATRA against APL.

## Figures and Tables

**Table 1:** ATRA-induced upregulation of several key proteins associated with calcium signaling.

Accession	Description	Log^2^FC	*p*-Value
P32455	Guanylate-binding protein 1	9.96	0
P33897	ATP-binding cassette sub-family D member 1	9.96	0
P48736	Phosphatidylinositol 4,5-bisphosphate 3-kinase catalytic subunit gamma isoform	9.96	0
Q9Y2J8	Protein-arginine deiminase type-2	9.96	0
O15162	Phospholipid scramblase 1	7.1	0.0034
O75923	Dysferlin	6.93	0.0039
P05109	Protein S100-A8	6.78	0.0043
P21980	Protein-glutamine gamma-glutamyltransferase 2	6.73	0.0045
P05362	Intercellular adhesion molecule 1	6.05	0.0072
P00813	Adenosine deaminase	5.82	0.0084
P37235	Hippocalcin-like protein 1	3.52	0.0420
P06702	Protein S100-A9	3.48	0.0430
P04271	Protein S100-B	3.44	0.0442
P08567	Pleckstrin	3.41	0.0451
P28676	Grancalcin	3.29	0.0493

## Data Availability

The mass spectrometry proteomics data have been deposited with the ProteomeXchange Consortium (http://proteomecentral.proteomexchange.org accessed on 21 November 2021) via the PRIDE partner repository ( http://www.ebi.ac.uk/pride accessed on 21 November 2021) [57] with the dataset identifier PXD026904, project Webpage: http://www.ebi.ac.uk/pride/archive/projects/PXD026904 accessed on 21 November 2021; FTP Download: ftp://ftp.pride.ebi.ac.uk/pride/data/archive/2021/11/PXD026904 accessed on 21 November 2021.

## Supplementary Materials

The following data are available online at https://www.mdpi.com/article/10.3390/cells10123364/s1, Supplementary Data 1: ATRA promotes Ca^2+^ entry in NB4 and Jurkat cells, Supplementary Data 2: ATRA-mediated autophagy involves calcium responses, Supplementary Data 3: GO terms associated with Ca^2+^ signaling. Supplementary Data 4: ATRA triggers AMPK activation in Hela cells through a Ca^2+^ -dependent mechanism.

## Abbreviations

2-APB	2-aminoethoxydiphenyl borate;
AMPK	AMP-activated protein kinase;
APL	acute promyelocytic leukemia;
ATRA	all-trans retinoic acid;
ATG	Autophagy-related gene;
BAPTA-AM	1,2-*Bis*(2-aminophenoxy)ethane-*N,N,N′,N*′-tetraacetic acid tetrakis(acetoxymethyl ester);
BTP2	*N*-(4-(3,5-*bis*(tri uoromethyl)-1H-pyrazol-1-yl)phenyl)-4-methyl-1,2,3-thiadiazole-5-carboxamide;
Ca^2+^	Calcium ions;
CAMKK2	calcium/calmodulin-dependent protein kinase kinase 2;
8-CPT-cAMP	8-(4-chlorophenylthio), 5’-cyclic adenosine monophosphate;
CRAC	calcium released activated channel; LC3-II, microtubule-associated protein 1 light chain 3-II;
ER	endoplasmic reticulum;
GO	gene ontology;
MGG	May-Grunwald Giemsa;
MFI	Median fluorescence intensity;
PI	propidium iodide;
ORAI1	ORAI calcium release-activated calcium modulator 1;
SERCA	sarco-/endoplasmic reticulum Ca^2+^-ATPase;
SOC	Store-operated calcium (SOC);
SOCE	store operated calcium entry;
sh	small hairpin;
SQSTM1	sequestosome 1;
STIM1	Stromal Interaction Molecule 1;
TG	thapsigargin.
TMRM	tetramethylrhodamine methyl ester;
MTORC1	mammalian target of rapamycin complex 1;
