# Supplementary material for: Targeting CAMKK2 and SOC Channels as a Novel Therapeutic Approach for Sensitizing Acute Promyelocytic Leukemia Cells to All-Trans Retinoic Acid
Source: Cells. 2021 Nov 30;10(12):3364. doi: 10.3390/cells10123364 (PMC8699360; doi:10.3390/cells10123364)
Supplement: Supplementary file 1 [file cells-10-03364-s001.zip › Supplementary data 1.pptx]

## Slide 1
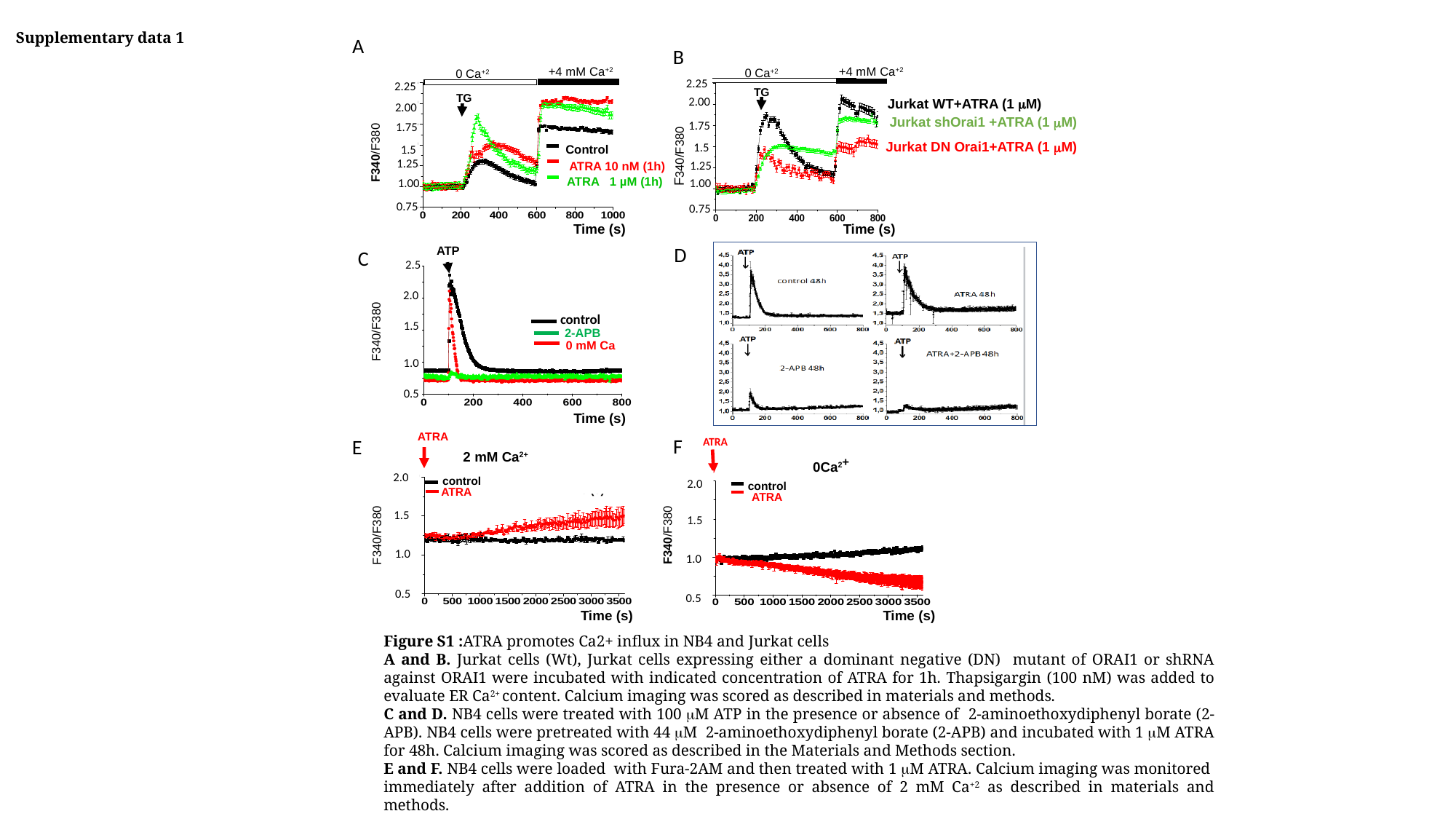

Supplementary data 1
A
B
+4 mM Ca+2
+4 mM Ca+2
0 Ca+2
TG
Control
ATRA 1 µM (1h)
F340/F380
ATRA 10 nM (1h)
Time (s)
0 Ca+2
Jurkat WT+ATRA (1 mM)
Jurkat shOrai1 +ATRA (1 mM)
F340/F380
Jurkat DN Orai1+ATRA (1 mM)
Time (s)
TG
D
ATP
control
2-APB
F340/F380
0 mM Ca
Time (s)
C
ATRA
E
2 mM Ca2+
control
ATRA
Time (s)
F340/F380
Time (s)
2.25
2.00
1.75
1.5
1.00
0.75
2.25
2.00
1.75
1.5
1.00
0.75
1.25
1.25
2.5
2.0
1.5
1.0
0.5
F
ATRA
0Ca2+
F340/F380
Time (s)
control
ATRA
2.0
1.5
1.0
0.5
2.0
1.5
1.0
0.5
Figure S1 :ATRA promotes Ca2+ influx in NB4 and Jurkat cells
A and B. Jurkat cells (Wt), Jurkat cells expressing either a dominant negative (DN) mutant of ORAI1 or shRNA against ORAI1 were incubated with indicated concentration of ATRA for 1h. Thapsigargin (100 nM) was added to evaluate ER Ca2+ content. Calcium imaging was scored as described in materials and methods.
C and D. NB4 cells were treated with 100 mM ATP in the presence or absence of 2-aminoethoxydiphenyl borate (2-APB). NB4 cells were pretreated with 44 mM 2-aminoethoxydiphenyl borate (2-APB) and incubated with 1 mM ATRA for 48h. Calcium imaging was scored as described in the Materials and Methods section.
E and F. NB4 cells were loaded with Fura-2AM and then treated with 1 mM ATRA. Calcium imaging was monitored immediately after addition of ATRA in the presence or absence of 2 mM Ca+2 as described in materials and methods.
