# Supplementary material for: Targeting CAMKK2 and SOC Channels as a Novel Therapeutic Approach for Sensitizing Acute Promyelocytic Leukemia Cells to All-Trans Retinoic Acid
Source: Cells. 2021 Nov 30;10(12):3364. doi: 10.3390/cells10123364 (PMC8699360; doi:10.3390/cells10123364)
Supplement: Supplementary file 1 [file cells-10-03364-s001.zip › Supplementary data 2.pptx]

## Slide 1
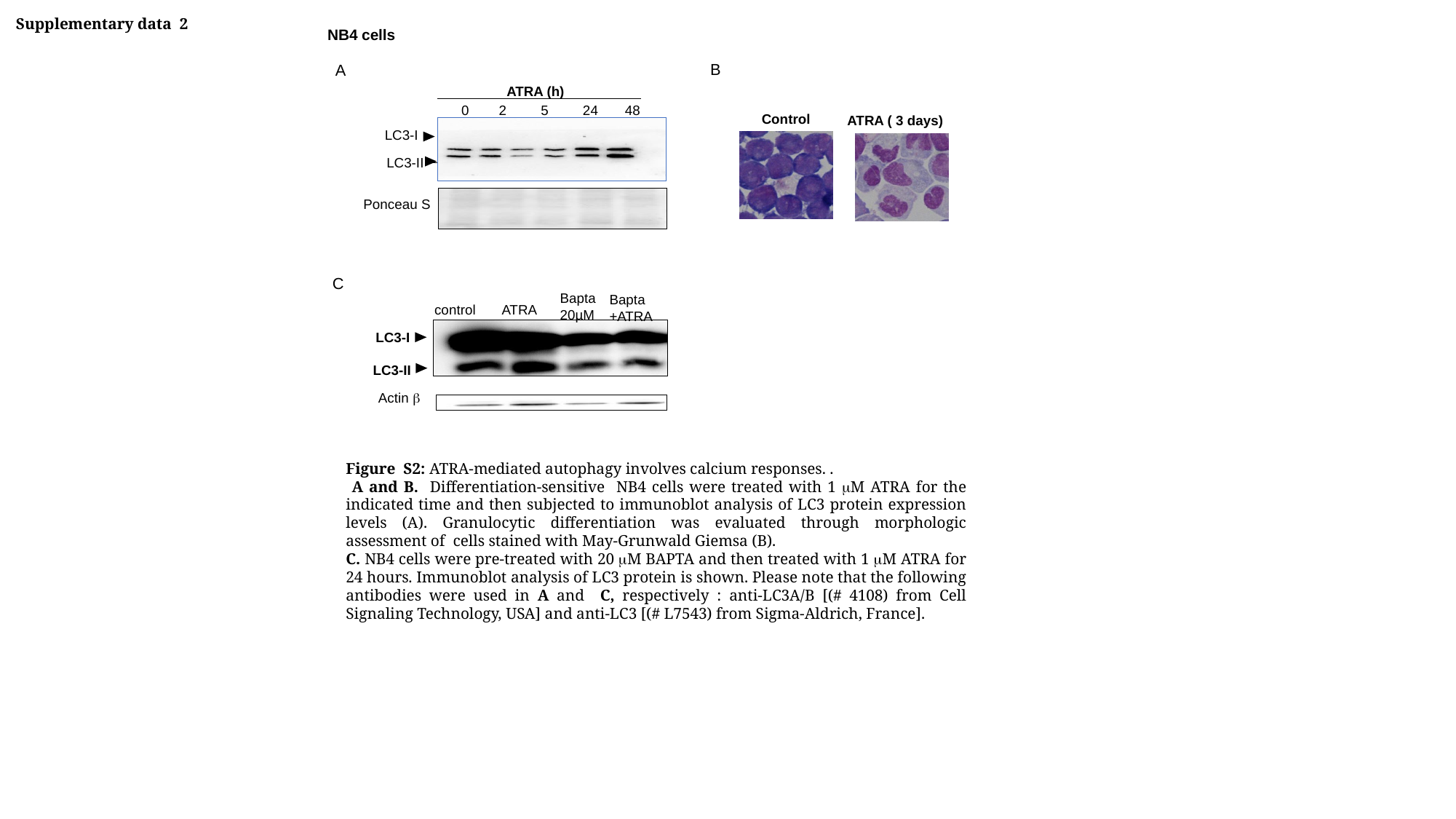

Supplementary data 2
NB4 cells
B
A
ATRA (h)
 0
 2 5 24 48
LC3-I
LC3-II
Ponceau S
Control
ATRA ( 3 days)
C
Bapta
20µM
Bapta
+ATRA
ATRA
control
 LC3-I
 LC3-II
Actin b
Figure S2: ATRA-mediated autophagy involves calcium responses. .
 A and B. Differentiation-sensitive NB4 cells were treated with 1 mM ATRA for the indicated time and then subjected to immunoblot analysis of LC3 protein expression levels (A). Granulocytic differentiation was evaluated through morphologic assessment of cells stained with May-Grunwald Giemsa (B).
C. NB4 cells were pre-treated with 20 mM BAPTA and then treated with 1 mM ATRA for 24 hours. Immunoblot analysis of LC3 protein is shown. Please note that the following antibodies were used in A and C, respectively : anti-LC3A/B [(# 4108) from Cell Signaling Technology, USA] and anti-LC3 [(# L7543) from Sigma-Aldrich, France].
