# Supplementary material for: Targeting CAMKK2 and SOC Channels as a Novel Therapeutic Approach for Sensitizing Acute Promyelocytic Leukemia Cells to All-Trans Retinoic Acid
Source: Cells. 2021 Nov 30;10(12):3364. doi: 10.3390/cells10123364 (PMC8699360; doi:10.3390/cells10123364)
Supplement: Supplementary file 1 [file cells-10-03364-s001.zip › Supplementary data 4.pptx]

## Slide 1
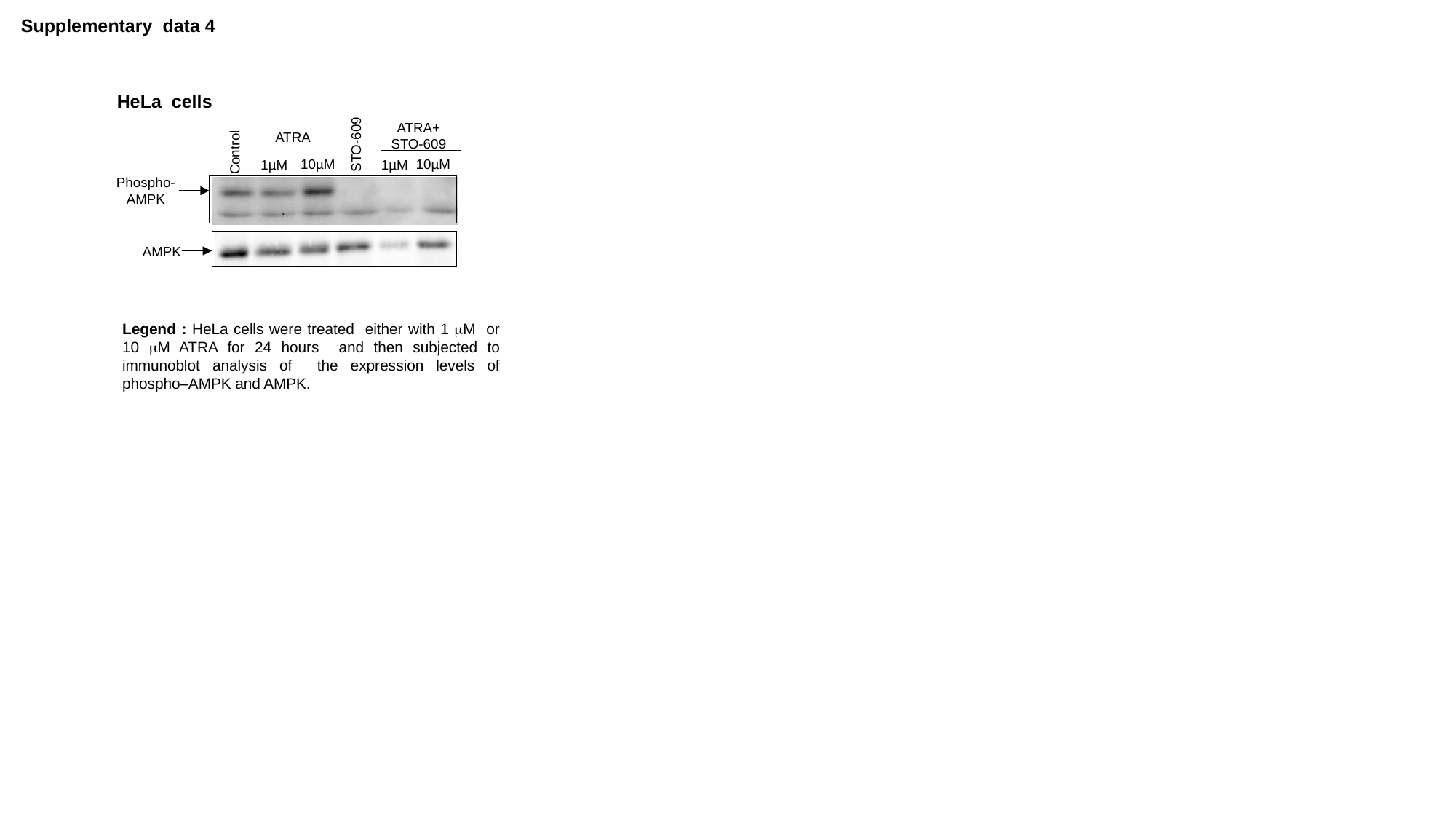

Supplementary data 4
HeLa cells
ATRA+
STO-609
STO-609
ATRA
Control
10µM
10µM
1µM
1µM
Phospho-AMPK
AMPK
Legend : HeLa cells were treated either with 1 mM or 10 mM ATRA for 24 hours and then subjected to immunoblot analysis of the expression levels of phospho–AMPK and AMPK.
